# Development of Time Series Models and Algorithms: Creep Prediction for Low-Carbon Concrete Materials

**DOI:** 10.3390/ma18133152

**Published:** 2025-07-03

**Authors:** Zhengpeng Zhou, Houmin Li, Keyang Wu, Jie Chen, Tianhao Yao, Yunlong Wu

**Affiliations:** 1 School of Civil Engineering, Architecture and the Environment, Hubei University of Technology, Wuhan 430068, China; 2 Innovation Demonstration Base of Ecological Environment Geotechnical and Ecological Restoration of Rivers and Lakes, Hubei University of Technology, Wuhan 430068, China; 3 Wuhan Construction Engineering Group Co., Ltd., Wuhan 430014, China

**Keywords:** low-carbon concrete, time-series models, time-dependent creep compliance, creep effect, optimization algorithm, machine learning optimization

## Abstract

In practical engineering applications, the use of low-carbon concrete materials is in line with the principles of sustainable development and helps to reduce the impact on the environment. Creep effects are particularly critical in the research on such materials. However, traditional characterization methods are time-consuming and often fail to account for the interactions of multiple factors. This study constructs a time-series database capturing the behavioral characteristics of low-carbon concrete materials over time. Three temporal prediction models—Artificial Neural Network (ANN), Random Forest (RF), and Long Short-Term Memory (LSTM) networks—were retrained for creep prediction. To address limitations in model architecture and algorithmic frameworks, an enhanced Adaptive Crowned Porcupine Optimization algorithm (ACCPO) was implemented. The improved performance of the ACCPO was validated using four diverse benchmark test functions. Post-optimization results showed remarkable improvements. For ANN, RF, and LSTM, single-metric accuracies increased by 20%, 19%, and 6%, reaching final values of 95.9%, 93.9%, and 97.8%, respectively. Comprehensive evaluation metrics revealed error reductions of 22.6%, 7.9%, and 8% across the respective models. These results confirm the rationality of the proposed temporal modeling framework and the effectiveness of the ACCPO algorithm. Among them, the ACCPO-LSTM time series model is the best choice.

## 1. Introduction

As a predominant material in civil engineering, concrete is extensively used in structures like bridges, roads, tunnels, and dams. However, the long-term performance of concrete in service, particularly its creep behavior, is a critical factor affecting structural durability and safety. For example, prestressed continuous rigid-frame bridges often show excessive long-term deflection and web cracking during prolonged use [1]. Concrete containment structures in nuclear power plants may undergo gradual performance deterioration during prolonged use [2]. In addition, the cracking of industrial concrete floors can lead to reduced load-bearing capacity and impaired structural durability [3]. These challenges highlight the need for systematic research on the creep characteristics of low-carbon concrete materials.

**Figure 1:**
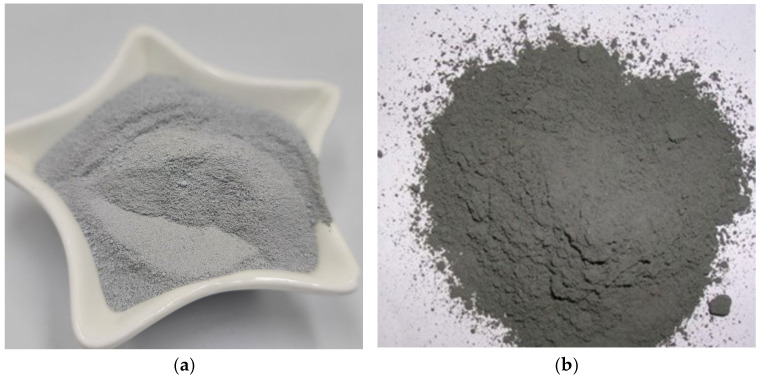
(**a**) Silica fume; (**b**) fly ash.

**Figure 2:**
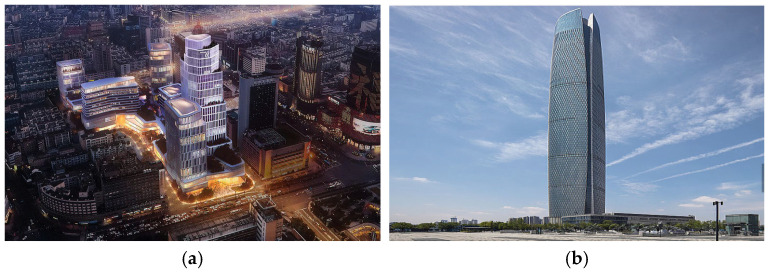
(**a**) Hangzhou Hanglong Plaza; (**b**) Wuhan Center Building.

The selection of low-carbon concrete materials, such as fly ash admixtures and silica fume, as a common low-carbon concrete material is driven by the need to promote sustainable development. As in Figure 1, these materials have the following characteristics: fine particle size, high specific surface area, and high chemical reactivity. Concrete mixed with an appropriate amount of silica fume has the property of significantly improving the compressive strength, durability and corrosion resistance of concrete [4,5,6,7]. For example, Hangzhou Hanglong Plaza (Figure 2a), Wuhan Center Building (Figure 2b) and other buildings use such materials, effectively reducing carbon emissions and practicing the concept of green low-carbon. However, compared to ordinary concrete, the creep behavior of low-carbon concrete materials remains unclear, especially under varying conditions, and the specific mechanisms influencing this creep behavior have not yet been fully elucidated [8,9,10]. Therefore, accurately predicting the creep behavior of low-carbon concrete materials has become an urgent challenge that needs to be addressed.

Traditional creep studies are based on extensive experimental data. These experiments typically require lengthy observation periods and are limited by the complexity of the experimental conditions [11]. To date, most international creep studies can be categorized as using nonlinear theoretical models. For example, Bu P, Li Y, Li Y, et al. employed fracture mechanics theory to analyze the actual energy release rate at crack tips in materials undergoing creep deformation [12]. This approach revealed the strain energy accumulation patterns in concrete microcracks under sustained loading, effectively demonstrating the coupling mechanism between concrete damage and creep. Internationally recognized prediction models include four versions proposed by the European Concrete Committee and the International Federation for Prestressing (CEB-FIP): the CEB-FIP (MC1970) model [13], the CEB-FIP (MC1978) model [13], the CEB-FIP (MC1990) model [14], and the FIB MC2010 model [15]. The American Concrete Institute (ACI) Committee 209 introduced the ACI-209R (1982) model [13] and the ACI-209R (1992) model [16] in 1982 and 1992, respectively. Subsequently, Professor Bažant and colleagues developed the B4 model [17] based on micro-prestressing solidification theory, incorporating comprehensive considerations of concrete strength, composition, and long-term creep behavior. Although these models progressively account for various factors influencing concrete creep, they exhibit certain limitations in predicting long-term deformation characteristics.

Recent advancements in data acquisition technologies and computational methodologies have propelled machine learning-based modeling approaches into increasing prominence within creep research. Machine learning techniques have emerged as effective tools for addressing the nonlinear behavior of concrete [18,19,20,21,22]. Notably, Taha et al. developed an artificial neural network (ANN) with a single hidden layer containing six neurons for masonry creep prediction. However, this study considered only four parameters and validated the model with a limited dataset of 14 samples, resulting in constrained generalizability [18]. Given that concrete creep represents typical time-series data, the integration of temporal modeling frameworks appears particularly promising. Thanh Bui-Tien et al. demonstrated the superiority of temporal models over conventional approaches through adaptive cells and deep learning methods in bridge damage assessment using time-series data [23]. Jian Liu et al. developed a multivariate time-series model for asphalt pavement rutting prediction, which outperformed comparative frameworks including ARIMAX, Gaussian process, and mechanistic–empirical (M-E) models [24]. Wang et al. established an LSTM network-based concrete creep model using experimental data, achieving satisfactory prediction accuracy. Nevertheless, this model neglected the influence of material properties and environmental factors on creep behavior [25]. These studies collectively indicate that temporal modeling architectures exhibit distinct advantages in processing time-dependent, nonlinear data with inherent noise through neural networks and deep learning paradigms. Their demonstrated effectiveness stems from their inherent ability to capture temporal dependencies and complex interaction patterns within sequential data structures.

In machine learning models, parameter configuration directly determines predictive performance, making parameter optimization particularly critical [26]. Optimization algorithms exhibit unique advantages and are extensively used for tuning machine learning parameters. The Crested Porcupine Optimizer (CPO), proposed in 2024 [27], represents a novel metaheuristic algorithm. It features a robust global search ability, rapid convergence, minimal parameter requirements, easy implementation, and synergistic compatibility with other algorithms. These characteristics have enabled its application across diverse fields such as water resources management [28] and geological exploration [29]. While standard CPO demonstrates notable merits, challenges persist, including local optima entrapment, parameter sensitivity, computational efficiency limitations, and restricted adaptability in dynamic environments. Addressing these issues remains a significant research frontier. Algorithmic enhancement strategies have proven effective in improving optimization performance. For example, an in-depth analysis of various methods for improving the Sine Cosine Algorithm (SCA) not only comprehensively summarizes its advantages and disadvantages, but also provides a broader study of meta-heuristic optimization algorithms [30]. Huang et al. introduced a simulated degradation mechanism to counteract the insufficient global search capacity in particle swarm optimization, achieving marked improvement in solution accuracy [31].

In summary, this study utilizes the characteristics of time series of low-carbon concrete material data, together with the consideration of multivariate variables such as material properties, environmental parameters and historical values, and establishes a time series deep learning prediction model based on the improvement of three machine learning models, namely, artificial neural networks (ANN), random forests (RF), and long- and short-term memory networks (LSTM), to predict creep. In order to improve the prediction ability of the model, four strategies are used to optimize and improve the CPO, and a new adaptive crown porcupine optimization algorithm (ACCPO) is established; the combination of the ACCPO and the time-series model greatly improves the prediction effect.

## 2. Materials and Methods

### 2.1. Database Description and Variable Analysis

The experimental data were collected from the NU database established by Professor Bažant at Northwestern University on the concrete creep and shrinkage NU database, and 1439 sets of creep test data were used as the basis [32]. The low-carbon concrete material data studied in this paper were compiled from this database.

Given the differences in parameter categories and test periods among various groups in the database, this study processed the database data to eliminate their impacts on model development:

1. In the description and analysis of the database, a unified coding rule is adopted for the selection of inputs and outputs and the handling of missing values;
2. For test groups of sufficient length in the database, due to the characteristics of concrete creep, which grows rapidly in the early stages and slowly in the later stages, data from the time period between 1 and 60 days were selected, and data less than 60 days were removed;
3. In processing and selecting good datasets, logarithmic transformations are applied to time. This transformation helps to achieve a more uniform distribution of creep time series data, thereby improving the predictive accuracy of the model;
4. Since the recording times of each data group are different, it is necessary to standardize the time intervals of each data group so that they can be easily input into the time series model. Because the data have been log-transformed, the recording times are approximately linear, so linear interpolation is used to unify the recording times of each data group;
5. Different input parameters have different dimensions and ranges. When using loss functions to calculate errors, features with larger scales play a decisive role in model training. Such input conditions can affect the results of data analysis and even cause the model results to fail to converge. To eliminate differences in dimensions between indicators, the data must be normalized.

Given that the B4 model is based on the widely accepted micro-prestressing solidification theory, it is reasonable to select the model’s input parameters according to this theory [33]. An analysis of the creep data in the database revealed that material properties such as concrete strength, elastic modulus, and mix ratio have the greatest influence on creep, followed by geometric characteristics and environmental factors like temperature and humidity. Based on the parameter selection of the B4 model [33] and the above analysis, 12 feature parameters were chosen as the model’s inputs, as presented in Table 1.

For variables f_c28_ and E_28_, if one is missing, the other can be estimated and supplemented using Equation (1) [34]. If both data points are missing, we remove the data.

(1) $$E_{28}=4734\sqrt{f_{c28}}$$

The unit of E_28_ and f_c28_ are MPa.

Because cement type is also a key factor in creep, the order of cement types was coded by the authors in order to incorporate this factor into the model. Cement types, including unknown, normal-setting R, rapid-setting RS, and slow-setting SL, are uniformly encoded according to the rules in Table 2.

The creep database for low-carbon concrete materials required for this study was obtained through the above-mentioned processing approach. Table 3 describes the minimum, maximum, and average values of input and output values in the database.

**Figure 3:**
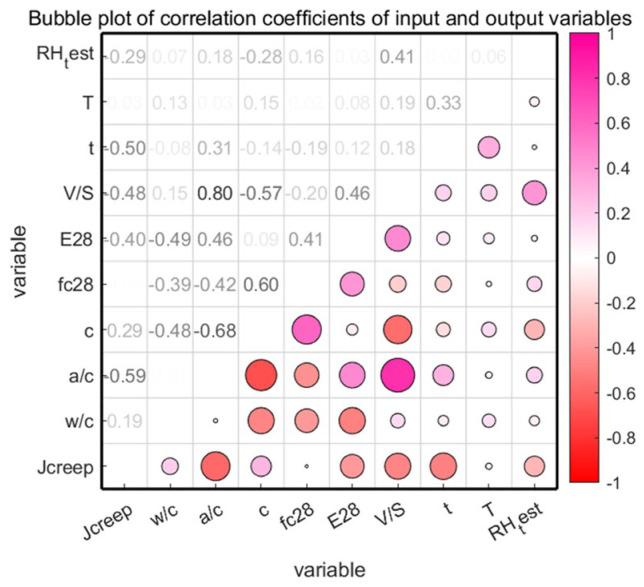
Heatmap of correlation coefficients.

**Figure 4:**
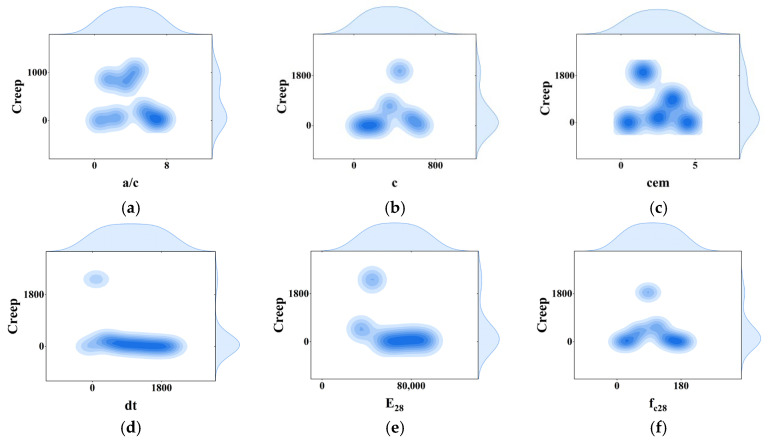

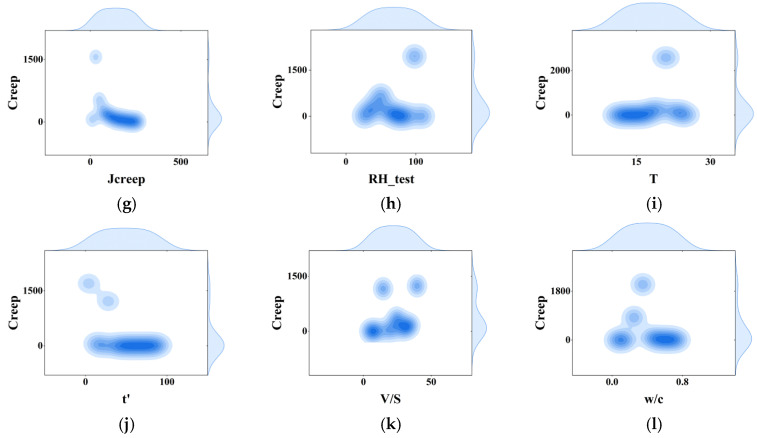
Joint distribution plot of input and output variables. (**a**) a/c and creep’s variable relationship; (**b**) c and creep’s variable relationship; (**c**) cem and creep’s variable relationship; (**d**) dt and creep’s variable relationship; (**e**) E_28_ and creep’s variable relationship; (**f**) f_c28_ and creep’s variable relationship; (**g**) Jcreep and creep’s variable relationship; (**h**) RH_test and creep’s variable relationship; (**i**) the variable relationship between T and creep; (**j**) the variable relationship between t’ and creep; (**k**) the variable relationship between V/S and creep; (**l**) the variable relationship between w/c and creep.

To explain the interdependencies among the model’s input variables, i.e., multicollinearity, we can consult [35]. Ideally, the correlation between variables should be less than 0.8 for the model to be considered a high-precision model [36]. A correlation coefficient heat map, as presented in Figure 3, illustrates the relationships between the input variables and the model output. Given the unique nature of time-series models and the need for unified coding rules, time points and cement types are not discussed. The heat map clearly shows that the impact of multicollinearity is relatively small. Additionally, Figure 4 shows the joint distribution of input and output variables, providing a clear visualization of the data ranges for these variables.

### 2.2. Optimization Algorithm for Crown Porcupines

The Crown Porcupine Optimizer (CPO) was proposed in 2024, inspired by the various defensive behaviors of the Crown Porcupine (CP) [27]. The crowned porcupine employs four defense mechanisms to counter different threats, ranging from the mildest to the most aggressive: visual, auditory, olfactory, and physical attacks. The first two defense mechanisms (visual and auditory) primarily reflect the exploratory behavior of the crowned porcupine, while the latter two (olfactory and physical attacks) reflect its exploitative behavior. In this algorithm, by simulating the four defense behaviors of the crowned porcupine, four zones are divided from outer to inner based on increasing aggressiveness to model defense behaviors. The outermost zone represents the first defense zone, implementing visual defense; the second defense zone implements auditory defense; the third defense zone implements olfactory defense; and the fourth defense zone implements physical attacks. The defense mechanisms in each zone are activated sequentially based on the threat level of the predator. The introduction of this mechanism helps accelerate the convergence speed of the algorithm while maintaining population diversity. The key technologies of this algorithm lie in population initialization, the technique of reducing the population in each iteration, and the four defense strategies.

For population initialization, the CPO algorithm is a search process that starts from a set of initial individuals. It can be expressed by Equation (2):

(2) $$\vec{X_{i}}=\vec{L}+\vec{r}\times \left(\vec{U}-\vec{L}\right)|i=1,2,3,\cdot ,\cdot ,\cdot ,N$$

in the equation, N represents the number of individuals, i.e., the population size, $\vec{x_{i}}$ is the i-th candidate solution in the search space, $\vec{L}$ and $\vec{U}$ are the lower and upper bounds of the search range, respectively, and $\vec{r}$ is a randomly initialized vector between 0 and 1.

For the cyclic population reduction (CPR) technique, some CPs are obtained from the population during the optimization process to accelerate the convergence speed, and then reintroduced into the population. This is used to determine the execution frequency during the optimization process, and is expressed mathematically as Equation (3):

(3) $$N=N_{min}+\left(N^{\prime }-N_{min}\right)*(1-(\frac{t\%\frac{T_{max}}{T}}{\frac{T_{max}}{T}}))$$

in the equation, N is the current population size, N_min_ is the minimum number of individuals in the newly generated population, N′ is the initial population size, t is the current number of function evaluations, T is the number of iterations, T_max_ is the maximum number of function evaluations, and % denotes the modulo operator.

For the four defense strategies:

1. First line of defense strategy

When the CP becomes aware of the predator, the predator has two choices: either continue approaching or move away. A random value is generated using a normal distribution to simulate these two options. When these random values are less than 1 or greater than −1, the predator moves closer to the CP; otherwise, the predator moves away from the CP. This can be defined as in Equation (4),

(4) $$\vec{{x_{i}}^{t+1}}=\vec{{x_{i}}^{t}}+T_{1}\times \left|2\times T_{2}\times \vec{{x^{t}}_{CP}}-\vec{{y_{i}}^{t}}\right|$$

Here, $\vec{{x_{i}}^{t+1}}$ and $\vec{{x_{i}}^{t}}\;$represent the positions of the predator at iterations t + 1 and t, respectively, $\vec{{x^{t}}_{CP}}$ is the optimal solution for function computation t, $\vec{{y_{i}}^{t}}$ is a vector generated between the current CP and a randomly selected CP from the population to represent the position of the predator at iteration t, where T_1_ is a random number based on a normal distribution, and T_2_ is a random value in the interval [0, 1]. The formula for calculating $\vec{{y_{i}}^{t}}$ is as shown in Equation (5),

(5) $$\vec{{y_{i}}^{t}}=\frac{\vec{{x_{i}}^{t}}+\vec{{x_{r}}^{t}}}{2}$$

where r is between [1, N], and *N* is the population size.

2. Second defense strategy.

In this strategy, the CP makes noise by emitting sounds to threaten predators. As predators approach, the porcupine’s sounds gradually increase in volume. This can be expressed by the following formula, shown in Equation (6),

(6) $$\vec{{x_{i}}^{t+1}}=(1-\vec{U_{1}})\times \vec{{x_{i}}^{t}}+\vec{U_{1}}\times (\vec{y}+T_{3}\times (\vec{{x^{t}}_{r1}}-\vec{{x^{t}}_{r2}}))$$

where r1 and r2 are two random values between [1, N], and T_3_ is a random value between 0 and 1.

3. Third defense strategy.

In this strategy, CP secretes foul-smelling gases to form a diffusion range that prevents predators from approaching. This can be expressed as in Equation (7),

(7) $$\vec{{x_{i}}^{t+1}}=(1-\vec{U_{1}})\times \vec{{x_{i}}^{t}}+\vec{U_{1}}\times (\vec{{x_{r1}}^{t}}+{S_{i}}^{t}\times (\vec{{x_{r2}}^{t}}-\vec{{x_{r3}}^{t}})-T_{3}\times \vec{\delta }\times \gamma_{t}\times {S_{i}}^{t})$$

Among these, r^3^ is [1, N], δ is a parameter used to control the search direction, defined by Equation (8), ${x_{i}}^{t}$ is the position of the i-th individual at iteration t, $\gamma_{t}$ is the defense coefficient defined by Equation (9), T_3_ is a random value in the interval [0, 1], and ${S_{i}}^{t}$ is the odor diffusion factor, defined by Equation (10), as shown below—

(8) $$\vec{\delta }=\left\{\begin{array}{c} +1,if\;\vec{rand}\leq 0.5\\ -1,Else \end{array}\right.$$

(9) $$\gamma_{t}=2\times rand\times {\left(1-\frac{t}{t_{max}}\right)}^{\frac{t}{t_{max}}}$$

(10) $${S_{t}}^{i}=\exp\left(\frac{f({x_{t}}^{i})}{\sum N_{k=1}f({x_{t}}^{k})+\varepsilon }\right)$$

where f(x_t_^i^) denotes the objective function value of the i-th individual at iteration t, ε is a small value used to avoid division by zero, $\vec{rand}$ is a vector containing random values generated between 0 and 1, wherein rand is a variable containing random numbers generated between 0 and 1, N is the total size, t is the current iteration number, and t_max_ is the maximum number of iterations.

4. Fourth defense strategy.

Finally, CP adopts a physical attack strategy. When the predator is very close, CP strikes it with short, thick feathers. At this point, the two objects collide violently, simulating a one-dimensional inelastic collision. This can be expressed by the following formula:

(11) $$\vec{{x_{i}}^{t+1}}=\vec{{x^{t}}_{CP}}+(\alpha (1-T_{4})+T_{4})\times (\delta \times \vec{{x^{t}}_{CP}}-\vec{{x_{i}}^{t}})-T_{5}\times \delta \times \gamma_{t}\times \vec{{F_{i}}^{t}}$$

where $\vec{{x^{t}}_{CP}}$ is the best solution obtained representing CP, $\vec{{x_{i}}^{t}}$ is the position of the i-th individual at iteration t, representing the predator at that position, α is the convergence speed factor of z, T4 and T5 are random values in the interval [0, 1], and $\gamma_{t}$ is the defense coefficient. Furthermore, $\vec{{F_{i}}^{t}}$ is the average force affecting the CP of the i-th predator.

### 2.3. Improving the Optimization Algorithm for Crown-Shaped Pigs

In order to minimize the occurrence of local optima, premature convergence, parameter sensitivity, and dynamic environment adaptability when solving the objective function, multiple strategic optimizations were performed on the conventional CPO algorithm. The specific steps are as follows.

#### 2.3.1. Logistic Chaotic Mapping Step Size Adjustment

The global search capability of the algorithm can be improved by chaotic mapping (Logistic chaotic mapping), which is characterized by traversal and non-repeatability to avoid falling into the local optimum [37]. Therefore, the step size (dynamic_step) in the original algorithm is changed from fixed or simply random to achieve a better balance between the exploration and development phases. The Logistic Chaos Mapping function expression is as follows:

(12) $$X_{n+1}=\mu x_{n}(1-x_{n})$$

in this context, μ is the control parameter, typically ranging from [0, 4], while x_n_ represents the value at the nth iteration, ranging from [0, 1]. When μ falls between 3.57 and 4, the Logistic map enters a chaotic state, at which point the generated sequence exhibits good randomness and ergodicity.

After adjusting the expression, the following are established:

(13) chaos_value=4×chaos_value×(1−chaos_value)

(14) dynamic_step=chaos_value×(1−t/Max_iterations)

in this context, chaos_value refers to the chaos value, and Max_iterations denotes the maximum number of iterations.

The adjustment improves the global search ability of the algorithm, avoids falling into local optimums, and produces sequences with good randomness and traversability.

#### 2.3.2. Dynamic Adjustment of Population Size

The dynamic stochastic wandering strategy refers to the iterative process of the optimization algorithm, according to the current search state and historical information, which involves dynamic adjustment to enhance the exploration ability of the algorithm in order to strengthen its ability to jump out of the local optimum [38]. The waste of computational resources caused by too large a population size can be avoided by dynamically adjusting the population size in this algorithm. The specific expression of dynamic stochastic wandering is as follows:

(15) $${X_{i}}^{t+1}={X_{i}}^{t}+step\times randn(0,1)$$

in this equation, ${X_{i}}^{t}$ denotes the position of the i-th individual in the t-th generation, step is the step size, and randn(0,1) denotes a random number from the standard normal distribution.

Based on the algorithm, we improve the initial expression by using Equations (16)–(18):

(16) cycle_length=Max_iterations/2

(17) phase=rem(t, cycle_length)/cycle_length

(18) New_Search_Agents=fix(N_min+(Search_Agents−N_min)×(1−phase×phase))

among them, New_Search_Agents is the new population size, N_min is the minimum population size, Search_Agents is the initial population size, phase is the parameter of the current stage, t is the current iteration number, and cycle_length is the cycle length.

The adjustment prevents premature convergence caused by too small a population size, and improves the efficiency and robustness of the algorithm.

#### 2.3.3. Adaptive Adjustment Strategy

Adaptive adjustment is a parameter adjustment strategy that allows the algorithm to automatically adjust its parameters based on the characteristics of the objective function [39]. In the algorithm, this strategy is applied to the probabilities of exploration and exploitation (exploration_prob and exploitation_prob) and the parameters alpha and Tf. First, the fixed probabilities of exploration and exploitation in the original algorithm are adjusted to probabilities that adaptively adjust with the number of iterations, as shown in Equations (19)–(22).

(19) exploration_prob=0.5×(1−t/Max_iterations)

(20) exploitation_prob=1−exploration_prob

(21) alpha=0.2×(1−t/Max_iterations)

(22) Tf=0.8×(1−t/Max_iterations)

After adjustment, it is helpful to conduct broader exploration in the early stages of the algorithm and undertake more in-depth development in the later stages.

#### 2.3.4. Boundary Handling

For boundary handling, algorithms such as particle swarm optimization [40] and genetic algorithms [41] encounter boundary handling issues during the optimization process. Furthermore, simplifying the boundary handling mechanism and reducing computational complexity are important in many practical applications, as complex boundary handling can slow down the algorithm and cause it to get stuck in a local optimum.

Therefore, in order to improve the stability and reliability of the algorithm, the complex boundary handling mechanism in the original algorithm was simplified to a more concise and effective method without affecting the functionality of the original algorithm. The simplified expression is as follows:

(23) X(i, :)=max(min(X(i, :), Upperbound), Lowerbound)

This adjustment allows the algorithm’s computational complexity to be reduced while ensuring that the search agent always remains within the valid search space.

### 2.4. Combine Implementation with Specific Steps

**Figure 5:**
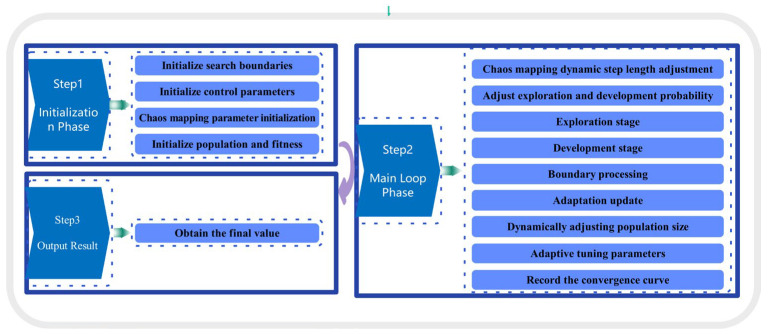
Specific steps of algorithm improvement.

The specific steps of the algorithm’s improvement are shown in Figure 5.

### 2.5. Test Function

In the study of optimization algorithms, to verify their performance improvements in the context of problems of varying complexity, various test functions are employed [42]. In this paper, to validate the performance of the improved CPO, we select four representative functions—the Sphere function, the Max function, the Step function, and the Sum of Absolute Values and Product function. Using the unique characteristics of these four functions, we conduct comparative experiments, with the test functions listed in Table 4. The test functions f1 and f2 in Table 4 are single-peak test functions used to assess the optimization accuracy of the optimization algorithm, while f3 and f4 are multi-peak test functions used to evaluate the algorithm’s ability to escape local optima. The setting of the ranges of these functions is based on the mathematical properties of each function, the actual constraints of the problem, and the different aspects of the optimization capabilities that need to be comprehensively evaluated to ensure a comprehensive and accurate assessment of the algorithm performance. This enables the test functions to comprehensively assess the performance of the optimization algorithms in different scenarios, for both global exploration challenges and local optimization tests. The optimization results of these four test functions are analyzed via aspects such as optimization accuracy, convergence speed, and stability.

**Figure 6:**
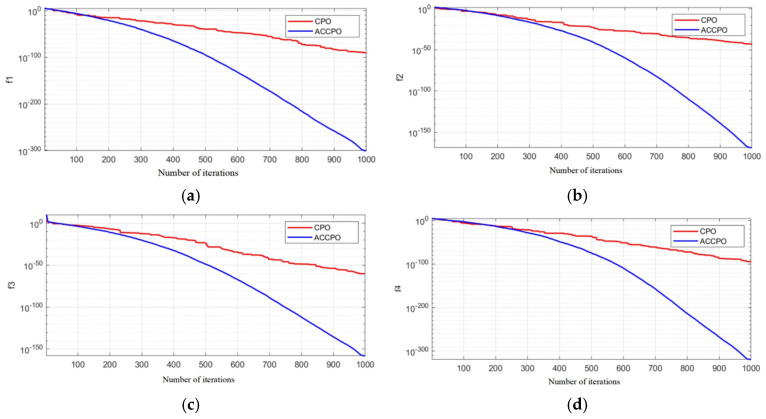
Optimization iteration curves for the 4 test functions. (**a**) f1 iteration curves, (**b**) f2 iteration curves, (**c**) f3 iteration curves, (**d**) f4 iteration curves.

In order to more intuitively compare the advantages of the algorithm in terms of convergence speed and optimization accuracy before and after optimization, the number of search agents (populations) is set to 100 by fixing the parameter settings, the maximum number of iterations is set to 1000, and the optimization algorithm optimizes the optimization iterative curves for the four test functions given in Figure 6, with the horizontal axis being the number of iterations, and the vertical axis being the optimal objective function value searched for in the algorithm over the successive iterations. The ACCPO objective function value curve of the algorithm decreases rapidly with the increase in the number of iterations, indicating that the optimization performance is greatly improved.

**Figure 7:**
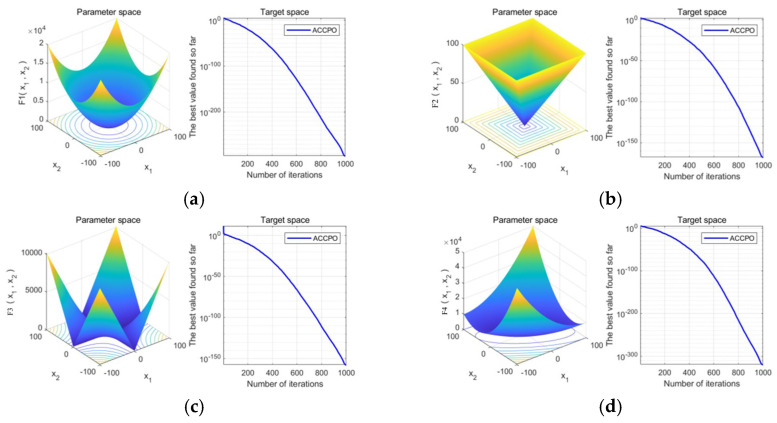
(**a**–**d**) The 3D plots of the four test functions of (f1–f4) and the convergence curves of the objective function values, respectively.

Then, by comparing the solutions of ACCPO in the 3D graphs derived from the test functions and deriving the optimal value of the objective function, the fixed parameter settings set the number of search agents (populations) to 100, and we set the maximum number of iterations to 1000, as shown in Figure 7 and Table 5. Combining Figure 7 and Table 5, it can be seen that the performance of the algorithm on these test functions is effectively improved by combining the other three strategies.

## 3. Machine Learning Models

**Figure 8:**
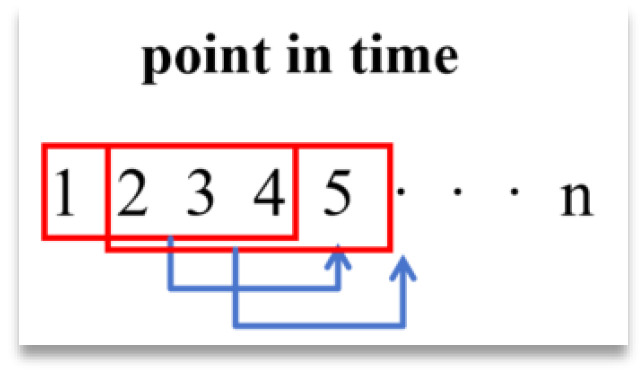
Sliding way within the same group.

Figure 8 represents a group with n time points, and the first four points are read each time to predict the next point, and so on to the nth point; i.e., the sliding window slides in a node-by-node sliding pattern within the same trial group.

**Figure 9:**
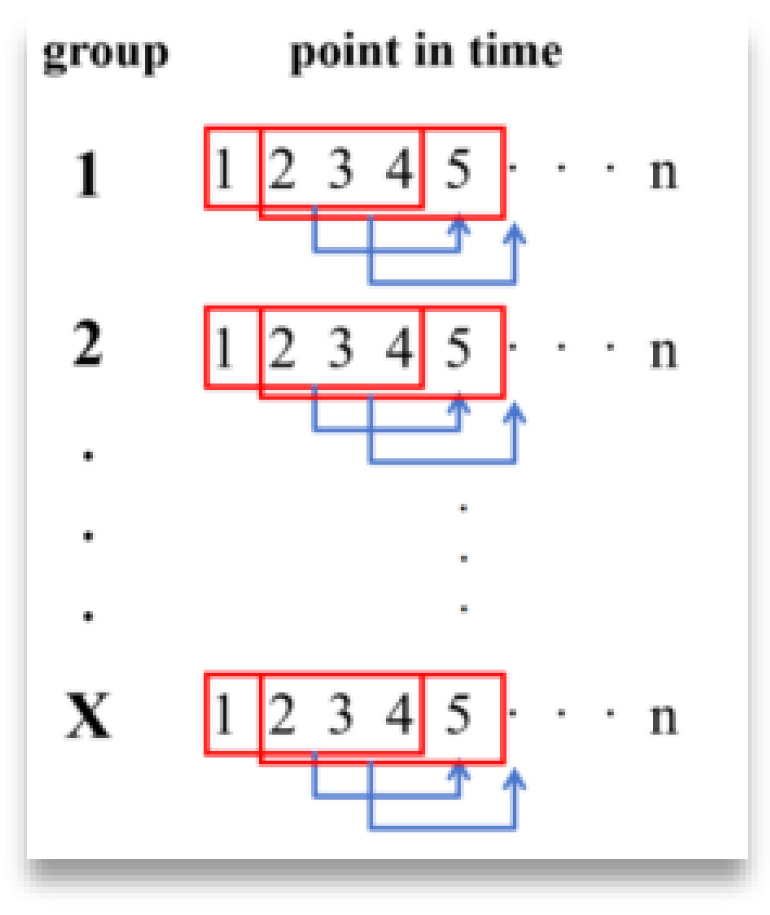
Different ways of sliding within groups.

The second case regards sliding within the different experimental groups in the way shown in Figure 9.

The sliding window in this figure will jump directly to the beginning of the next sample after the training of the previous set of data is completed, starting the traversal of the second set of samples, and so on, until all the data have been trained.

### 3.1. ANN Model

The Artificial Neural Network (ANN) is not designed to take into account the properties of time series per se [43]. The inputs and outputs of an ANN are independent, and it is not able to capture temporal dependencies as automatically as a specialized LSTM. Therefore, a sliding window is almost essential when seeking to process time series with ANN. Through sliding windows, we can transform the time series data into a format that the ANN can understand, and train time series models in this way. An ANN usually consists of input, hidden and output layers. ANN neural networks are widely used in model prediction due to their powerful nonlinear mapping capabilities [44].

Finding the optimal neuron in an ANN model is very important. Therefore, in this manuscript, we first use an empirical formula to determine a range of values, and then use an algorithm to optimize and fix the optimal value. The expression is

(24) $$h=\sqrt{m+n}+a$$

where m represents the number of nodes in the input layer, and n represents the number of nodes in the output layer, a ∈ (1, 10).

**Figure 10:**
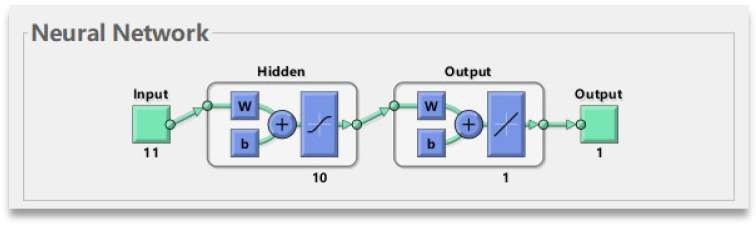
ANN neural network.

Secondly, AS weights and biases are optimized using the established ACCPO. Figure 10 shows the input, hidden and output layers that the ANN neural network has.

### 3.2. RF Model

Random Forest is an integrated learning method that works by voting or averaging results to obtain a final prediction [45]. It is not specifically designed to be used for time series data per se, but when dealing with time series problems, the model can also be adapted to achieve the desired results [46].

**Figure 11:**
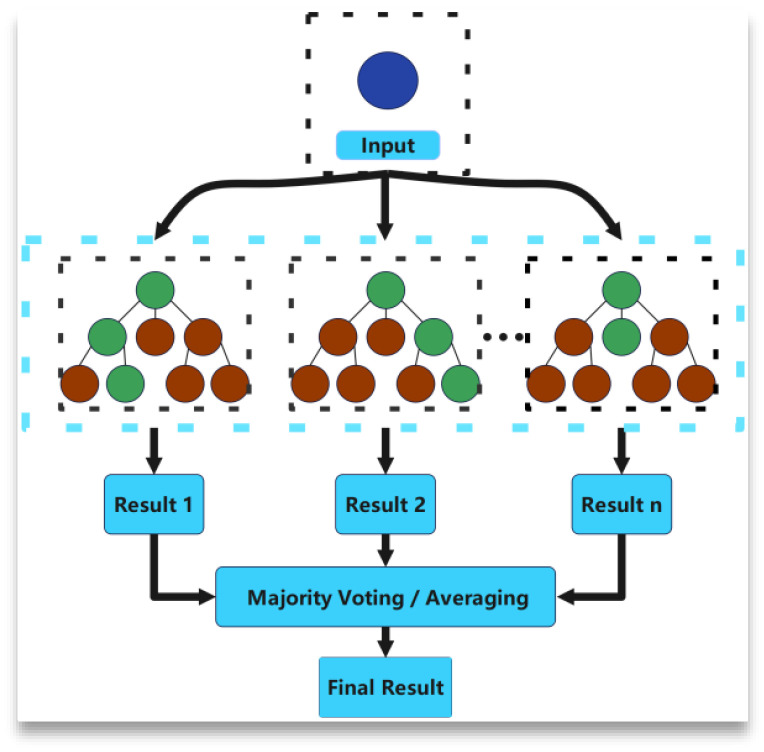
Random Forest model.

The key parameters of Random Forest mainly include the number of trees (the more decision trees, the better the performance of the model usually is, but the computational cost will increase accordingly) and the number of randomly selected features (the number of randomly selected features when each node is split; usually, the number of randomly selected features is equal to the square root or logarithmic value of the total number of features). In general, the selection of the number of features affects the bias and variance of the model. Figure 11 shows the RF model.

The performance of RF depends largely on the setting of its hyperparameters, such as the number of decision trees (n_estimators), the maximum depth of decision trees (max_depth), etc., in order to improve the accuracy and efficiency of the time series prediction. So by adjusting the hyperparameters such as the number of trees, the maximum depth of the tree, etc., to make the model optimal, the hyperparameters in the established ACCPO optimized Random Forest model can help the RF to achieve better performance in the time series prediction task.

### 3.3. LSTM Model

For the Long Short-Term Memory recurrent neural network (LSTM), the model itself is usable for time series prediction, as compared to the machine learning models mentioned above [47]. For the study of LSTM, the weights and biases in the model, the setting of important hyperparameters, and the selection of the sliding window have a greater impact on the model. In this study, a brand new LSTM is constructed for training by processing the dataset.

**Figure 12:**
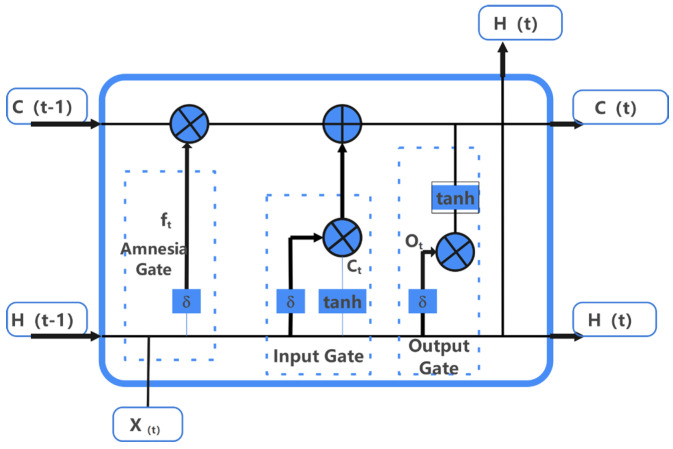
LSTM structure.

Regarding weights and biases in LSTM, LSTM recurrent neural networks introduce memory units (memory states) to control data transmission between hidden layers. A memory unit in an LSTM network consists of three gate structures: input gates, forget gates, and output gates. The input gates determine how much of the current input is retained in the current unit state; the forget gates determine how much of the previous unit state is retained in the current unit state; and the output gates determine how much of the current unit state is output. The structure is shown in Figure 12.

The structural functions of each gate of the LSTM network are shown below in Equations (25)–(27),

(25) $$I_{t}=\delta (w_{i1}x_{t}+W_{i2}h_{t-1}+b_{i})$$

(26) $$f_{t}=\delta (w_{f1}x_{t}+W_{f2}h_{t-1}+b_{f})$$

(27) $$O_{t}=\delta (w_{O1}x_{t}+W_{O2}h_{t-1}+b_{O})$$

where I_t_, f_t_ and O_t_ are the vector values of the input, forgetting and output gates of a node of the LSTM neural network at time t, respectively; x_t_ is the input at time t; b_i_, b_f_ and b_o_ are the corresponding bias values of the gate structures, respectively; w_1_ is the connection weight between the input node and the hidden node; w_2_ is the connection weight between the hidden node and the output node; h_t−1_ is the output at time t−1, which represents the hidden state (hidden state) of the LSTM. h_t−1_ is the output at time t−1, representing the hidden state of the LSTM.

For the optimization of weights and bias, we use Adam’s algorithm. Adam combines the advantages of two optimization algorithms, the AdaGrad algorithm [48] and RMSProp algorithm [49]. The first-order moment estimation and second-order moment estimation of the gradient are considered comprehensively by these, and different values of the learning rate are determined based on the results of the moment estimation, with the following expressions:

(28) $$m_{t}=\beta_{1}m_{t-1}+(1-\beta_{1})g_{t}$$

(29) $$v_{t}=\beta_{2}v_{t-1}+(1-\beta_{2}){g_{t}}^{2}$$

here, m_t_ and v_t_ are the first-order moment estimates and second-order moment estimates of the current gradient; g_t_ is the current gradient value; β_1_ and β_2_ are the coefficients.

Usually the values of m_t_ and v_t_ are corrected for bias, and the corrected Adam’s method expression is shown in the following:

(30) $$\theta_{t+1}=\theta_{t}-\frac{\eta }{\varepsilon +\sqrt{\overset{\wedge }{v_{t}}}}\overset{\wedge }{m_{t}}$$

This can be styled as

(31) $$\overset{\wedge }{m_{t}}=\frac{m_{t}}{1-{\beta_{1}}^{t}}$$

(32) $$\overset{\wedge }{v_{t}}=\frac{v_{t}}{1-{\beta_{2}}^{t}}$$

Finally, when using LSTM models for time series prediction, selecting and optimizing hyperparameters is crucial for model performance. The hyperparameters that need to be optimized include the number of hidden layer nodes, the learning rate, and the batch size.

### 3.4. Model Evaluation Indicators

#### 3.4.1. Single Indicator

In this study, root mean square error (RMSE), mean absolute error (MAE) and coefficient of determination (R^2^) are used to evaluate the performance of the model. R^2^ is mainly used to measure the correlation between the actual values and the predicted values. The closer the R^2^ is to 1, the smaller the MAE is, and the higher the model accuracy is. The following are the mathematical expressions for the three evaluation metrics:

(33) $$R^{2}=\frac{\sum_{k=1}^{N}(q_{0,k}-\bar{q_{0}})(q_{t,k}-\bar{q_{t}})}{\sqrt{\sum_{k=1}^{N}(q_{0,k}-\bar{q_{0}}{)}^{2}\sum_{k=1}^{N}(q_{t,k}-\bar{q_{t}}{)}^{2}}}$$

(34) $$MAE=\frac{1}{N}(\sum_{K=1}^{N}\left|\frac{q_{0,t}-q_{t,k}}{q_{0,k}}\right|)$$

(35) $$RMSE=\sqrt{\frac{1}{N}\sum_{k=1}^{N}(q_{0,k}-q_{t,k}{)}^{2}}$$

N in this equation denotes the number of samples; q_0_ denotes the actual value; $\bar{q_{0}}$ denotes the actual average value; q_t_ denotes the output value; $\bar{q_{t}}$ denotes the output mean, k∈<1,N>.

#### 3.4.2. Composite Indicators

In order to compare the prediction performances of different types of machine learning prediction models, this paper unifies the above three single statistical indexes (Equations (33)–(35)) into one comprehensive index for analysis, i.e., the Synthesis Performance Index (SPI) [50], as shown in Equation (36).

(36) $$SPI=\frac{1}{N}\sum_{j=1}^{N}\frac{P_{j}-P_{\min,j}}{P_{\max,j}-P_{\min,j}}$$

where N is the number of selected statistical indicators used to measure the prediction performance. In this paper, N = 3 because R^2^, RMSE and MAE are selected. In addition, P_j_ is the jth statistical parameter, and at the same time, P_max,j_ and P_min,j_ are the maximum and minimum indicators of the selected jth statistical parameter in the set of values of the machine learning model used, respectively. As can be seen from Equation (36), the size of the SPI value is distributed between [0, 1], and in terms of the overall prediction performance, when the value of SPI is closer to 0, it indicates that the performance of the machine learning prediction model represented by it is better, and vice versa, when the value of SPI is closer to 1, it indicates that the effectiveness of the machine learning prediction model represented by it is worse. In this paper, in terms of prediction performance, the SPI obtained from different types of machine learning training will be given the distribution of the advantages and disadvantages of the prediction effect of the model according to the size of its value.

## 4. Model Training Results

### 4.1. ACCPO-ANN Model

Based on the characteristics of the database and ANN, a three-layer feedforward ACCPO-ANN model was constructed. The range of the number of hidden layer neurons was obtained using Equation (24). The optimal value of 11 was obtained by optimizing the model using the ACCPO algorithm. In addition to these basic parameters, other relevant coefficients need to be determined, such as the maximum number of iterations and the learning rate. The parameters of the ACCPO-ANN model after hyperparameter optimization and global optimization are shown in Table 6.

Table 7 presents the evaluation metrics results of the three ANN models. After introducing the ACCPO algorithm, the predictive capability of the ANN models was significantly improved. The ACCPO-ANN model outperformed the CPO-ANN and traditional ANN models in both the training and testing datasets, achieving an R^2^ value of 0.9510 in explaining data variance and demonstrating excellent performance in reducing prediction errors (RMSE and MAE). Related studies indicate that if the R^2^ value is higher than 0.9, the model can be considered excellent [51]. Therefore, the ACCPO-ANN model is the most outstanding.

**Figure 13:**
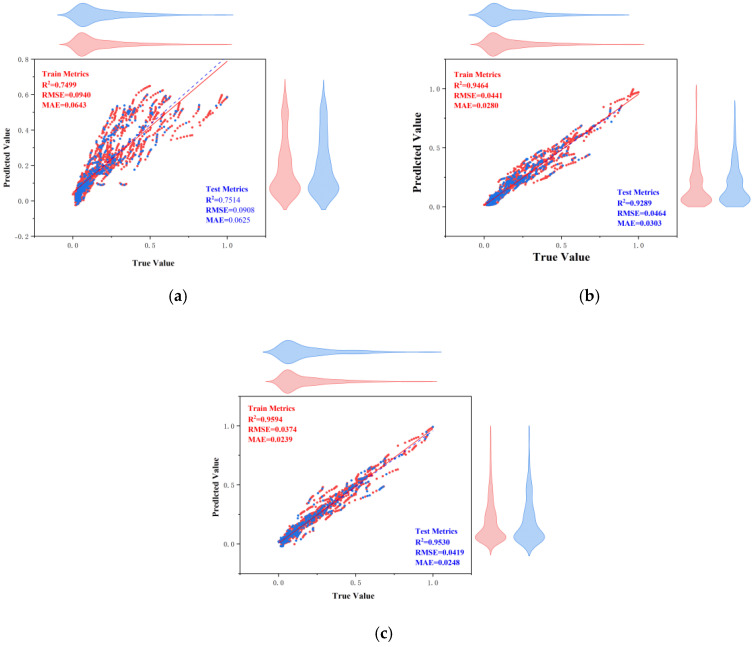
Scatter plot of regression analysis of actual and predicted values of the model: (**a**) represents ANN, (**b**) represents CPO-ANN, and (**c**) represents ACCPO-ANN.

**Figure 14:**
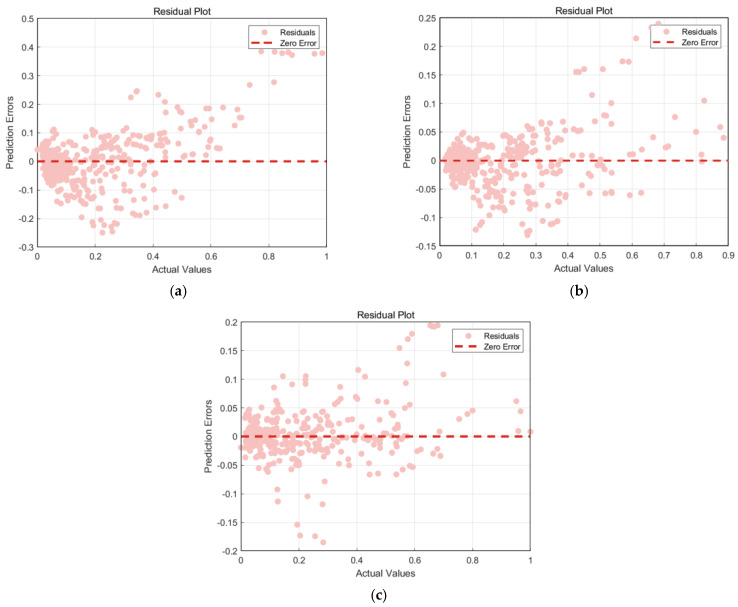
Comparison of residuals of model testing sets: (**a**) represents ANN, (**b**) represents CPO-ANN, and (**c**) represents ACCPO-ANN.

Figure 13 shows the regression analysis scatter plot comparing the actual values and predicted values of the three ANN models. The points represent data points, and the red dashed line indicates the ideal prediction line. Figure 14 shows the residual plots of the three models. The pink points represent residuals, and the red dashed line indicates the zero-error line. Combining the analysis of the two figures, we see that the data points of the traditional ANN model are generally distributed along the ideal prediction line, but there are deviations, and the residuals have a large fluctuation range. Although it can capture the overall trend of the data, the prediction accuracy in some areas is poor; the data points of the CPO-optimized ANN model are more closely clustered around the ideal prediction line, especially in the region where the actual values are small, and the residuals have a significantly reduced fluctuation range, with higher prediction accuracy for smaller values; the ACCPO-optimized ANN model shows high prediction accuracy in both smaller and larger value regions, with residuals almost concentrated near the zero-error line and a very small fluctuation range. This indicates that ACCPO successfully optimizes the ANN model, and the hybrid algorithm is effective.

### 4.2. ACCPO-RF Model

The combination of algorithms and RF is intended to optimize the number of random forest decision trees, leaf nodes, and the number of splitting features. The parameter configuration of the ACCPO-RF model is shown in Table 8.

Through the analysis of Table 9, we see that the RF model performs better on the test set than on the training set, indicating that the model is more effective in terms of the time series effect, but we also see that the optimization algorithm has a positive impact on the model performance, and that through optimization via the mixture of algorithms, the model’s performance is also improved by a small margin.

**Figure 15:**
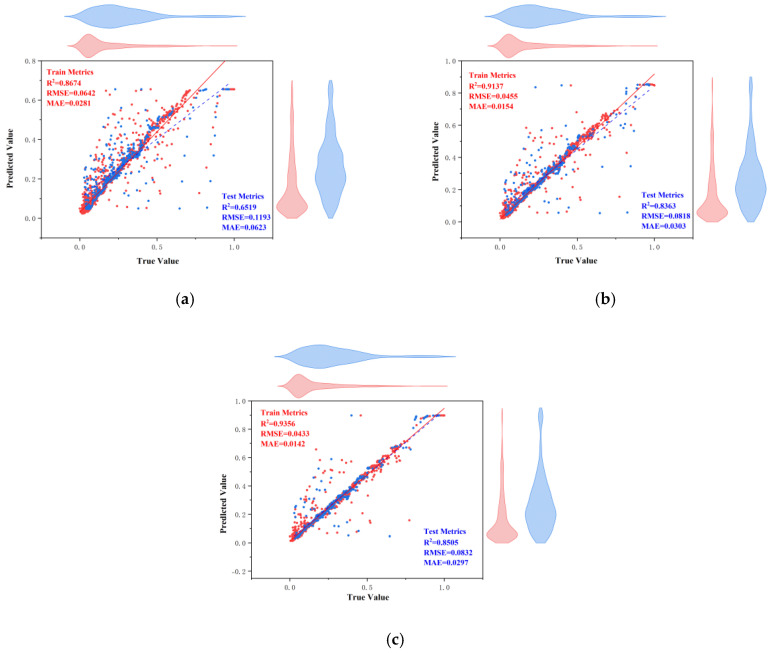
Scatter plot of regression analysis of actual and predicted values of the model: (**a**) represents RF, (**b**) represents CPO-RF, and (**c**) represents ACCPO-RF.

**Figure 16:**
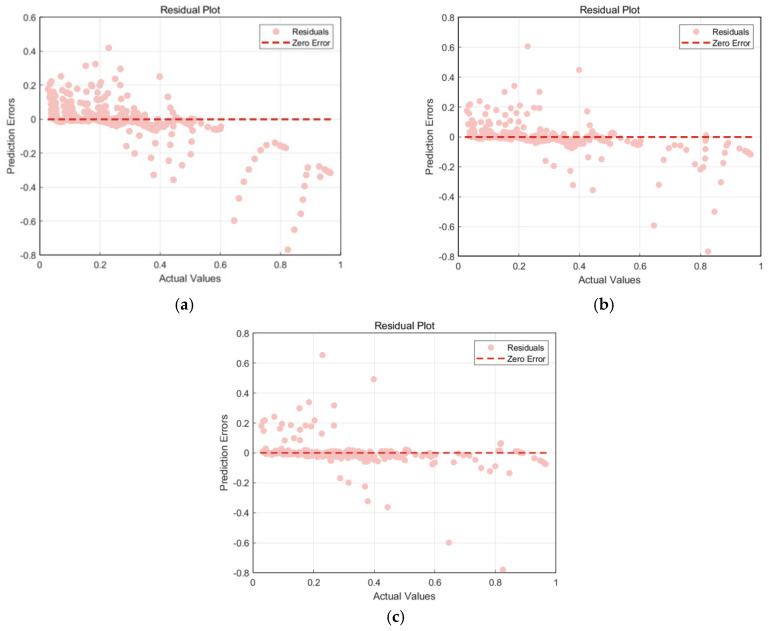
Comparison of residuals of model testing sets: (**a**) represents RF, (**b**) represents CPO-RF, and (**c**) represents ACCPO-RF.

By analyzing Figure 15 and Figure 16, it can be observed that as the model is optimized, the range of residuals gradually narrows, particularly in regions with higher actual values, where the fluctuation range of residuals significantly decreases, indicating that the model’s prediction error gradually decreases. The correlation between predicted values and actual values gradually increases, with predicted values becoming more concentrated and closer to the ideal prediction line, indicating that the model’s predictive capability is gradually improving. The ACCPO-RF model outperforms the other two models in terms of fitting ability and generalization ability.

### 4.3. ACCPO-LSTM Model

In this study, we delve into the strategy for the optimization of LSTM for the task of time series prediction. The weights and biases, sliding window, and some hyperparameters are optimized to achieve higher prediction accuracy. The parameters of the ACCPO-LSTM model are configured in Table 10.

Table 11 shows the performance metrics of the ACCPO-LSTM model on different datasets. The ACCPO-LSTM model achieves better performance on both the training set and the test set compared to the traditional LSTM model and the CPO-LSTM model, indicating that it has obvious advantages in prediction accuracy. These results fully verify the effectiveness of the proposed sliding window optimization setting and hyperparameter tuning strategy.

**Figure 17:**
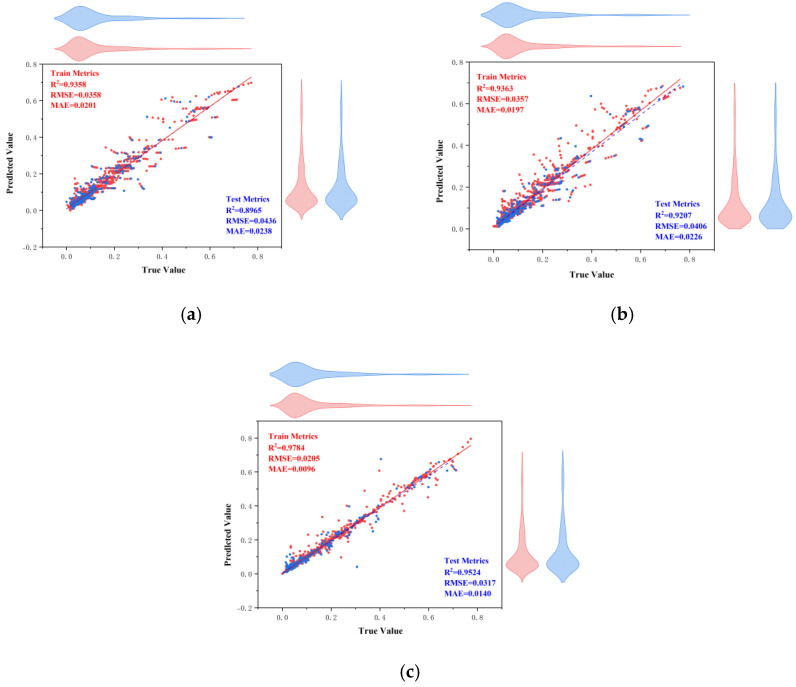
Scatter plot of regression analysis of actual and predicted values of the model: (**a**) represents LSTM, (**b**) represents CPO-LSTM, and (**c**) represents ACCPO-LSTM.

**Figure 18:**
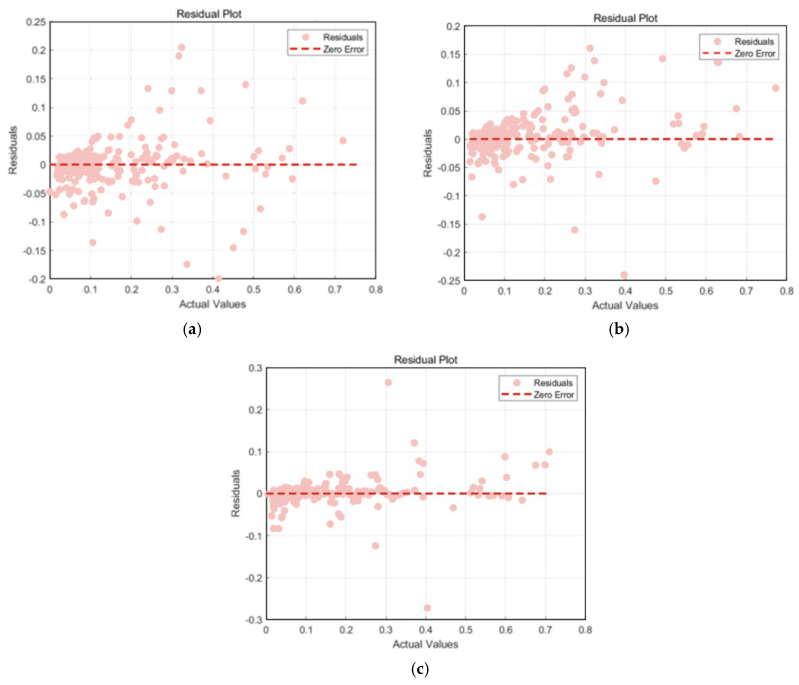
Comparison of residuals of model testing sets: (**a**) represents LSTM, (**b**) represents CPO-LSTM, (**c**) represents ACCPO-LSTM.

The graph of Test_Set_Actual_vs_Predicted in Figure 17 clearly demonstrates the high degree of consistency between the model’s predicted and actual values in terms of the overall trend and fluctuation patterns. The model is able to accurately capture and restore both the short-term fluctuations and long-term trends of the data, which fully reflects its strong ability to capture various types of features in time series data. From the Test_Residual_Plot in Figure 18, we see that the residual distribution of the ACCPO-LSTM model is relatively uniform and random, which indicates that the model fits the time series data adequately and there is no obvious systematic bias. Meanwhile, the absolute values of the residual values are generally small, indicating that the difference between the predicted and actual values of the model is small, which further validates the high prediction accuracy of the model.

## 5. Results

We divide the nine different models into training sets and test sets to obtain two evaluation metric summary tables, as shown in Table 12 and Table 13.

**Figure 19:**
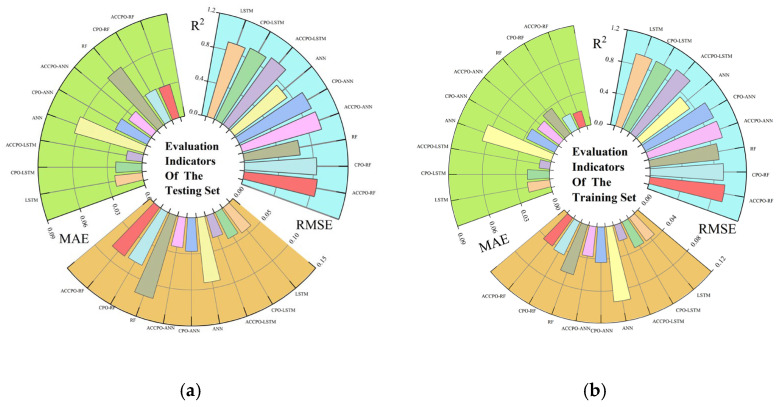
Three evaluation metrics corresponding to the 9 models: (**a**) representing the training sets, and (**b**) representing the testing sets.

**Figure 20:**
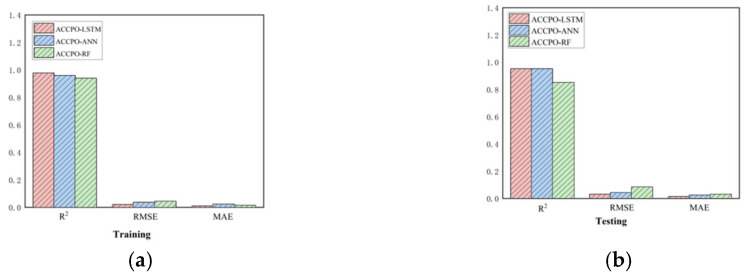
Three evaluation metrics for the ACCPO optimization model: (**a**) represents the training sets, and (**b**) represents the testing sets.

- Firstly, looking at Section 2.5 Test Function, combined with Figure 4, Table 5 shows that after the initial verification of the test function, the effect of the CPO algorithm following gradual optimization to ACCPO is further improved, implying that the improvement of the algorithm is successful.
- Secondly, the performances of the nine models on the training and test sets were compared using three evaluation metrics, as shown in Figure 19. From this, it can be seen that it is feasible to use the CPO algorithm for model performance enhancement. From this, it can also be concluded that the effects of the optimized algorithm ACCPO are further enhanced compared to CPO, thus verifying that the optimization of the algorithm is successful. Further, the ACCPO-LSTM model performed the best overall—on the training set, it achieved an R^2^ of 0.9784, an RMSE of 0.0205, and an MAE of 0.0096, surpassing all other models. On the test set, it maintained high accuracy, with an R^2^ of 0.9524, an RMSE of 0.0317, and an MAE of 0.014.
- Thirdly, based on three evaluation metrics, we can see the effect of training the ACCPO algorithm in order to enhance the ANN, RF and LSTM models on the training and test sets, as shown in Figure 20. The ACCPO-LSTM model exhibits the best performance on both the training and test sets, achieving the highest R^2^ and the lowest RMSE and MAE. Thus, it is evident that this model surpasses the other two models.

**Figure 21:**
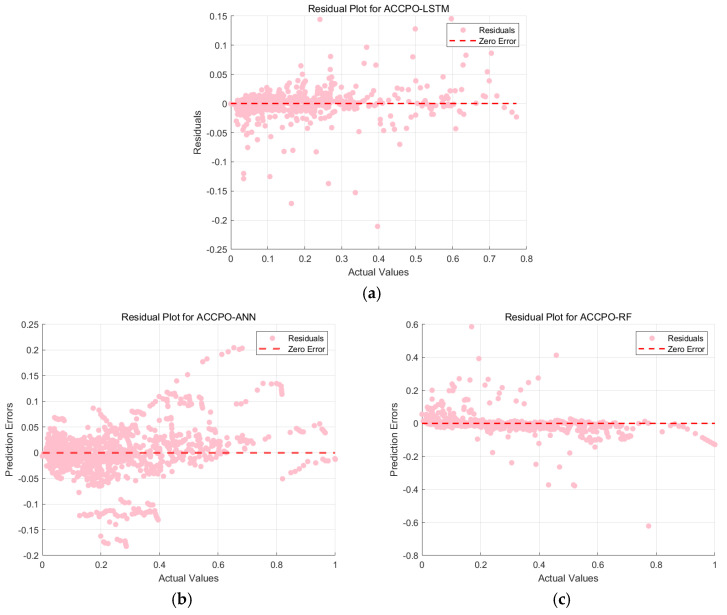
Residual analysis results of the three models’ training sets: (**a**) represents ACCPO-LSTM, (**b**) represents ACCPO-ANN, and (**c**) represents ACCPO-RF.

**Figure 22:**
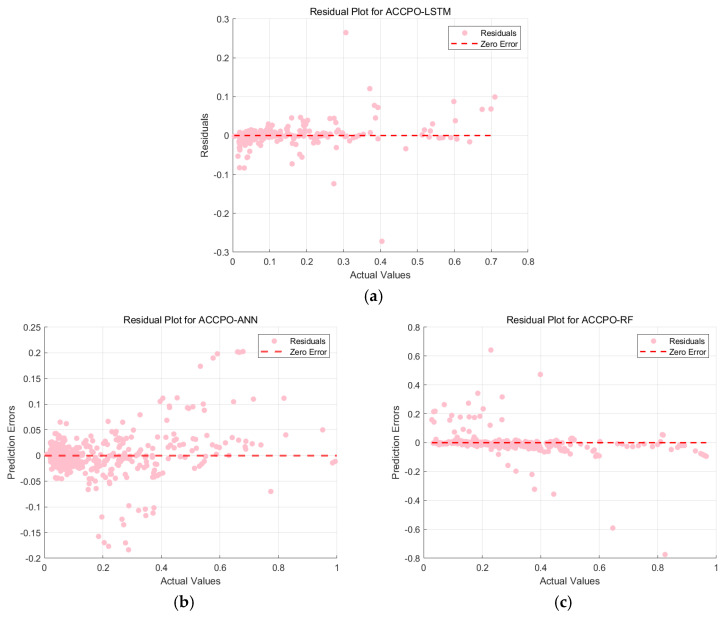
Residual analysis results of the three models on the testing sets: (**a**) represents ACCPO-LSTM, (**b**) represents ACCPO-ANN, and (**c**) represents ACCPO-RF.

This is then combined with the residual plots of the training and test sets, as shown in Figure 21 and Figure 22. The residuals of the ACCPO-LSTM model are more tightly clustered around the horizontal axis, indicating a closer alignment between predicted and actual values. Thus, residual analysis confirms that the ACCPO-LSTM model possesses the strongest predictive capability.

Thus, by analyzing the three evaluation indicators, as well as performing the residual analysis, it is possible to draw the following conclusion: the LSTM model enhanced by the ACCPO algorithm performs the best.

**Figure 23:**
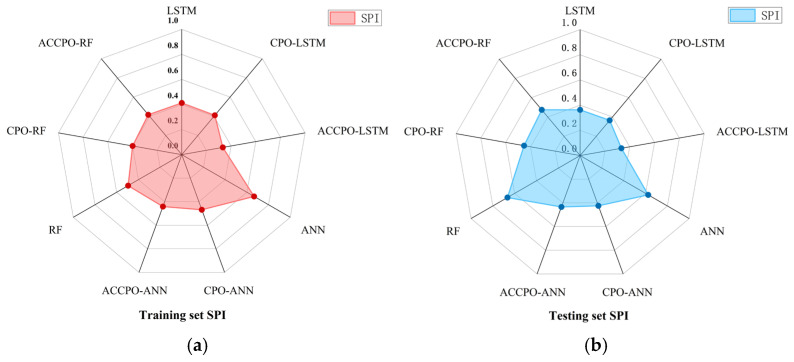
Radar plots of SPI pairs: (**a**) represents the training sets, and (**b**) represents the testing sets.

- Finally, a comparative analysis of the model’s composite indicators allows for a clearer and more intuitive comparison, as shown in Figure 23. Since the SPI value ranges from 0 to 1, a smaller value indicates better overall model predictability. The radar chart clearly shows that the ACCPO-LSTM model achieves the smallest SPI value across the entire machine learning model range, indicating its superior overall predictive performance.

Overall, with the above four results, it can be concluded that the optimization of the algorithm is successful. The ACCPO optimization method performed well across multiple models, significantly enhancing their predictive capabilities, particularly for LSTM and ANN. Further, while RF exhibited poor training set performance, its test set performance improved following ACCPO optimization.

## 6. Conclusions

This study investigates the creep of low-carbon concrete materials using machine learning techniques.

- The original algorithm was improved and enhanced by adding additional strategies, and its effectiveness was first tested using representative benchmark functions. The results show that the improved algorithm performed significantly better than the original algorithm;
- By modifying the source codes of three models (ANN, RF, and LSTM) and using them to train time series data, the results show that it is feasible to train the three models directly, but the results are generally not very good;
- The model is optimized using CPO and the proposed ACCPO, and the optimized model is used to train the data to improve the model’s performance and achieve the best training results;
- The predictive abilities of the models were evaluated using single indicators (R^2^, RMSE, MAE) and comprehensive indicators (SPI). The results show that the performances of the models optimized by ACCPO were improved, with ACCPO-LSTM being the optimal model;
- The results of this study indicate that time series models demonstrate significantly superior computational performance compared to other types of models when studying research subjects with time-dependent characteristics (such as creep). Therefore, in studies involving building materials with such time-dependent characteristics, it is recommended to prioritize the use of time series models to obtain optimal computational results. Finally, this study still has limitations, such as the selection of only four unique test functions for validation during the test function verification process. Future studies should consider expanding the number of test functions.

## Figures and Tables

**Table 1:** Selected input parameters.

Identification Number	Notation	Significance
A1	dt	Loading duration for creep, days.
A2	Jcreep	The historical creep compliance is expressed as 10^−6^/MPa.
A3	w/c	Water-cement ratio (by weight).
A4	a/c	Aggregate-cement ratio (by weight).
A5	c	Cement content, kg/m^3^
A6	cem	Cement type, including unknown, normal setting R, rapid setting RS, and slow setting SL.
A7	f_c28_	Average cylinder strength at 28 days, MPa.
A8	E_28_	Mean Young’s modulus at 28 days, MPa.
A9	V/S	Volume-surface area ratio, mm.
A10	t’	Age t’ during creep loading, days.
A11	T	Ambient temperature, °C.
A12	RH_test	Relative humidity of the environment, % (99 means a sealed specimen, 100 means storage in water, 101 steam, 85 moist)

**Table 2:** Encoding rules for cement types.

Cement Type	Unknown	Normal-Setting R	Rapid-Setting RS	Slow-Setting SL
Encoding Rule	0	1	2	3

**Table 3:** Statistical analysis of variables.

	Min	Max	Average
dt	0	4166.67	170.05
Jcreep	16.8	270.6	55.88
w/c	0.2	0.61	0.32
a/c	1.27	7.41	3.56
c	266	595	439.52
cem	1	3	1.66
f_c28_	43	136	91.87
E_28_	32,500	50,800	42,959.47
V/S	12	37	25.31
t’	0.66	91	14.17
T	18	23	19.99
RH_test	40	101	83.37

**Table 4:** Four test functions.

Functional	Dimension	Range of Values	Single Peak/Multiple Peaks
$f_{1}(x)=\sum_{i=1}^{n}x_{i}^{2}$	30	[−100, 100]	single peak
$f_{2}(x)=\underset{i}{\max}\left|x_{i}\right|$	30	[−100, 100]	single peak
$f_{3}\left(x\right)={\sum_{i=1}^{n}\left(\sum_{j=1}^{i}x_{j}\right)}^{2}$	30	[−10, 10]	multiple peaks
$f_{4}(x)=\sum_{i=1}^{n}\left|x_{i}\right|+\prod_{i=1}^{n}\left|x_{i}\right|$	30	[−100, 100]	multiple peaks

**Table 5:** Optimal values of the objective function found by the 4 test functions.

Functional	CPO	ACCPO
f1	1.2268 × 10^−96^	1.3146 × 10^−296^
f2	6.0520 × 10^−61^	8.4366 × 10^−169^
f3	9.6280 × 10^−53^	1.1896 × 10^−158^
f4	3.8164 × 10^−100^	2.1828 × 10^−320^

**Table 6:** Model parameters of ACCPO-ANN.

Parameters	Settings
Popsize	30
Maxgen	100
Activation function	tansig
Training function	trainlm
Epochs	48
Learning rate	0.01
Minimum performance gradient	1 × 10^−6^
Maximum validation failure	6

**Table 7:** Comparison of results of ANN model.

		R^2^	RMSE	MAE
ANN	training set	0.7499	0.0940	0.0643
	test set	0.7514	0.0908	0.0625
CPO-ANN	training set	0.9464	0.0441	0.0280
	test set	0.9289	0.0464	0.0303
ACCPO-ANN	training set	0.9594	0.0374	0.0239
	test set	0.9530	0.0419	0.0248

**Table 8:** Model parameters of ACCPO-RF.

Parameters	Settings
NumTrees	100
MinLeafSize	15
NumPredictorsToSample	6
OOBPrediction	on
OOBPredictorImportance	on
MinParentSize	2
MaxNumSplits	50

**Table 9:** Comparison of results of RF models.

		R^2^	RMSE	MAE
RF	training set	0.8674	0.0642	0.0281
	test set	0.6519	0.1193	0.0623
CPO-RF	training set	0.9137	0.0455	0.0154
	test set	0.8363	0.0818	0.0303
ACCPO-RF	training set	0.9396	0.0433	0.0142
	test set	0.8505	0.0832	0.0297

**Table 10:** Model parameter settings for ACCPO-LSTM.

Parameters	Settings
numFeatures	11
numRespones	1
maxEpochs	300
miniBatchSize	128
Search_Agents	30
Max_iterations	50
LearnRate	0.01
HiddenUnits	223
WindowSize	7

**Table 11:** Comparison of results for LSTM models.

		R^2^	RMSE	MAE
LSTM	training set	0.9358	0.0358	0.0201
	test set	0.8965	0.0436	0.0238
CPO-LSTM	training set	0.9363	0.0357	0.0197
	test set	0.9207	0.0406	0.0226
ACCPO-LSTM	training set	0.9784	0.0205	0.0096
	test set	0.9524	0.0317	0.0140

**Table 12:** Prediction performances of nine different models used on the training set.

	R^2^	RMSE	MAE	SPI
LSTM	0.9358	0.0358	0.0201	0.413
CPO-LSTM	0.9363	0.0357	0.0197	0.41
ACCPO-LSTM	0.9784	0.0205	0.0096	0.333
ANN	0.7499	0.094	0.0643	0.667
CPO-ANN	0.9464	0.0441	0.028	0.468
ACCPO-ANN	0.9594	0.0374	0.0239	0.441
RF	0.8674	0.0642	0.0281	0.495
CPO-RF	0.9137	0.0455	0.0154	0.399
ACCPO-RF	0.9396	0.0433	0.0142	0.416

**Table 13:** Prediction performances of nine different models used on the test set.

	R^2^	RMSE	MAE	SPI
LSTM	0.8965	0.0436	0.0238	0.363
CPO-LSTM	0.9207	0.0406	0.0226	0.365
ACCPO-LSTM	0.9524	0.0317	0.014	0.333
ANN	0.7514	0.0908	0.0625	0.624
CPO-ANN	0.9289	0.0464	0.0303	0.424
ACCPO-ANN	0.953	0.0419	0.0248	0.434
RF	0.6519	0.1193	0.0623	0.666
CPO-RF	0.8363	0.0818	0.0303	0.452
ACCPO-RF	0.8505	0.0832	0.0297	0.474

## Data Availability

The original contributions presented in this study are included in the article. Further inquiries can be directed to the corresponding author.
